# Efficacy and safety of praziquantel plus artemisinin-based combinations versus praziquantel in the treatment of Kenyan children with *Schistosoma mansoni* infection: open-label, randomized, head-to-head, non-inferiority trial

**DOI:** 10.1128/aac.00739-24

**Published:** 2024-12-19

**Authors:** Charles O. Obonyo, Vincent O. Were, Peter Wamae, Erick M. O. Muok

**Affiliations:** 1 Centre for Global Health Research, Kenya Medical Research Institute 118982 , Kisumu, Kenya; The Children's Hospital of Philadelphia, Philadelphia, Pennsylvania, USA

**Keywords:** schistosomiasis, praziquantel, artesunate, children, mefloquine, amodiaquine, sulfalene, dihydroartemisinin

## Abstract

Praziquantel alone is insufficient for the control of schistosomiasis due to poor efficacy against juvenile worms and increasing concerns about the risk of drug resistance. We compared the efficacy and safety of praziquantel combined with four different artemisinin-based combinations to praziquantel alone in treating *Schistosoma mansoni* infection in Kenyan children. In this randomized, open-label, five-arm, head-to-head, non-inferiority trial, children (aged 9–15 years) with *S. mansoni* infection according to duplicate Kato Katz thick smears from a stool sample in the Mwea irrigation scheme of central Kenya, were enrolled. Participants were randomly assigned (1:1:1:1:1) via a computer-generated block randomization procedure to receive a single oral dose of praziquantel (PZQ) (40 mg/kg/day) alone or in combination with a 3-day course (4 mg/kg of artesunate) of artesunate plus sulfalene-pyrimethamine (As + SP), artesunate plus amodiaquine (As + AQ), artesunate plus mefloquine (As + MQ) or dihydroartemisinin-piperaquine (DHAP). Laboratory technicians were masked to treatment allocation, but participants, clinicians, and study nurses were not. The primary outcomes were the cure rate and frequency of adverse events, which were assessed 6 weeks after treatment in the available case population using a per-protocol analysis. The non-inferiority margin was set at −10% for the risk difference in cure rates between combination therapy and PZQ alone. Between 12 September 2018 and 11 January 2019, 540 participants were assigned to receive PZQ alone (*n* = 108), PZQ plus As + SP (*n* = 108), PZQ plus As + AQ (*n* = 108), PZQ plus As + MQ (*n* = 108), or PZQ plus DHAP (*n* = 108). Primary outcome data were available for 523 (96.9%) participants. The cure rate was 82.5% (85/103) in PZQ alone, 81.7% (85/104) in PZQ plus As + SP, 76.2% (80/105) in PZQ plus As + AQ, 88.7% (94/106) in PZQ plus As + MQ, and 85.7% (90/105) in PZQ plus DHAP arm. Non-inferiority was declared for PZQ plus As + MQ (difference 6.2 [95% confidence interval: −3.3 to 15.6]) and PZQ plus DHAP (3.2 [−6.7 to 13.1]) but not for PZQ plus As + SP (−0.8 [−11.2 to 9.6]) or PZQ plus As + AQ (−6.3 [−17.3 to 4.6]). Adverse events were reported by 26% (138/540) of participants, including abdominal pain, headache, and vomiting. There were no serious adverse events. Alternatives to praziquantel should include praziquantel plus artesunate-mefloquine or praziquantel plus dihydroartemisinin-piperaquine. However, further multicentre trials are needed in different epidemiological settings and population groups to confirm these findings.

**CLINICAL TRIALS:**

This study is registered with the Pan-African Clinical Trials Registry under PACTR202001919442161.

## INTRODUCTION

Schistosomiasis, a neglected parasitic disease caused by trematode worms of the *Schistosoma* genus*,* is a major global health issue. The three main schistosome species responsible for human infections are *Schistosoma mansoni*, *S. haematobium*, and *S. japonicum* (1). Over 90% of the 250 million people infected with schistosomes reside in sub-Saharan Africa, where S. *mansoni* and *S. haematobium* are prevalent (2). The global burden of schistosomiasis is estimated at 1.4–3.3 million disability-adjusted life years annually (3). There is a large geographical overlap between malaria and schistosomiasis, especially in sub-Saharan Africa. The greatest burden of schistosomiasis is in preschool and school-aged children, with consequences that include anemia, school absenteeism, impaired child growth, physical fitness, and impaired cognitive and intellectual development (4). Other population groups at high risk include adults with occupations that involve contact with infested water, such as fishermen, car washers, sand harvesters, irrigation workers, farmers, and women doing domestic chores.

Praziquantel is the only drug recommended for the control and treatment of schistosomiasis, as it is active against all schistosome species that infect human populations, is administered as a single oral dose, is affordable, and is generally safe (5). However, praziquantel is only active against adult schistosome worms and is ineffective against the juvenile stages (schistosomulae) (6). Partly for this reason, praziquantel does not achieve a cure rate of 100%, and re-infection is frequent (7). Despite these limitations, mass administration of praziquantel has resulted in substantial reductions in schistosomiasis-associated morbidity and mortality (8). Schistosomiasis is one of the neglected tropical diseases set for elimination as a public health problem by 2030. However, reliance on a single drug for extensive mass drug administration has raised concerns over the potential drug pressure and risk of developing drug resistance. While evidence of clinical resistance is not yet established, laboratory and community studies have shown reduced schistosome susceptibility to praziquantel (9, 10). Consequently, new broad-spectrum anti-schistosomal targeting both adult and juvenile worms is urgently needed. Combination therapy, which involves administering two or more drugs with different mechanisms of action, is a promising strategy for delaying the development of praziquantel resistance. The drug development pipeline for schistosomiasis is currently empty. Drug repurposing is an optimal novel approach to overcome the cost and time limitations of developing new drugs for schistosomiasis by exploring new indications for approved medicines currently used for other conditions (11). Possible candidates for combination therapy for schistosomiasis include praziquantel plus antimalarials, such as the artemisinin derivatives (12).

The artemisinin derivatives, which are highly effective against malaria, have also shown activity against juvenile schistosome worms (13, 14). These differences in drug action with praziquantel provide a basis for combining praziquantel with artemisinin derivatives to enhance praziquantel efficacy and prevent drug resistance development. Experimental studies with animal models have provided evidence supporting this approach, showing that combining praziquantel and artemisinin derivatives is more effective than any of the medicines alone (15, 16). Similarly, a systematic review summarising the available evidence from exploratory studies in human participants has suggested the superior efficacy of praziquantel plus artemisinin derivatives against schistosomiasis (17). However, the evidence on the efficacy of combination therapy comprising praziquantel and artemisinin-based combination treatments (ACTs) compared to praziquantel alone is scanty and mixed (18, 19). In a small study, praziquantel plus artesunate-mefloquine showed no benefit in treating *S. haematobium,* while in a large trial, praziquantel plus dihydroartemisinin-piperaquine was highly effective in treating *S. mansoni* (18, 19). The World Health Organization (WHO) has approved at least five ACTs for treating uncomplicated malaria (20). However, to our knowledge, no published study has performed a head-to-head comparison of the effect of different combination therapies comprising ACT and praziquantel in treating schistosomiasis. This study aimed to establish that combination therapy is as safe and effective as praziquantel.

The primary objective of this proof-of-concept, phase III, open-label, five-arm, head-to-head, non-inferiority randomized controlled study was to compare the parasitological cure rates and safety between praziquantel alone and praziquantel combined with four different artemisinin-based combinations in children with intestinal schistosomiasis. The secondary objectives were to assess the cumulative cure and egg reduction rates by 12 weeks after treatment. The study tested the hypothesis that combination therapy is not inferior to praziquantel alone in treating schistosomiasis. A non-inferiority margin was set at −10 percentage points for the risk difference in cure rates between combination therapy and PZQ alone, which was similar to a margin used in a previous similar study (21) and also considered clinically equivalent. In this study, four ACTs were selected for repurposing because they are widely available for the treatment of uncomplicated falciparum malaria, are co-formulated, are administered once daily, have a well-established safety profile, and have previously been evaluated for the treatment of schistosomiasis but yielded inconclusive results (18, 19, 22–27). The selected ACTs include artesunate plus sulfalene-pyrimethamine, artesunate plus amodiaquine, artesunate plus mefloquine, or dihydroartemisinin-piperaquine. The multi-arm trial design was adopted to enable us to efficiently compare the different combination therapies with a single control (praziquantel) group in the same trial.

## RESULTS

**Fig 1:**
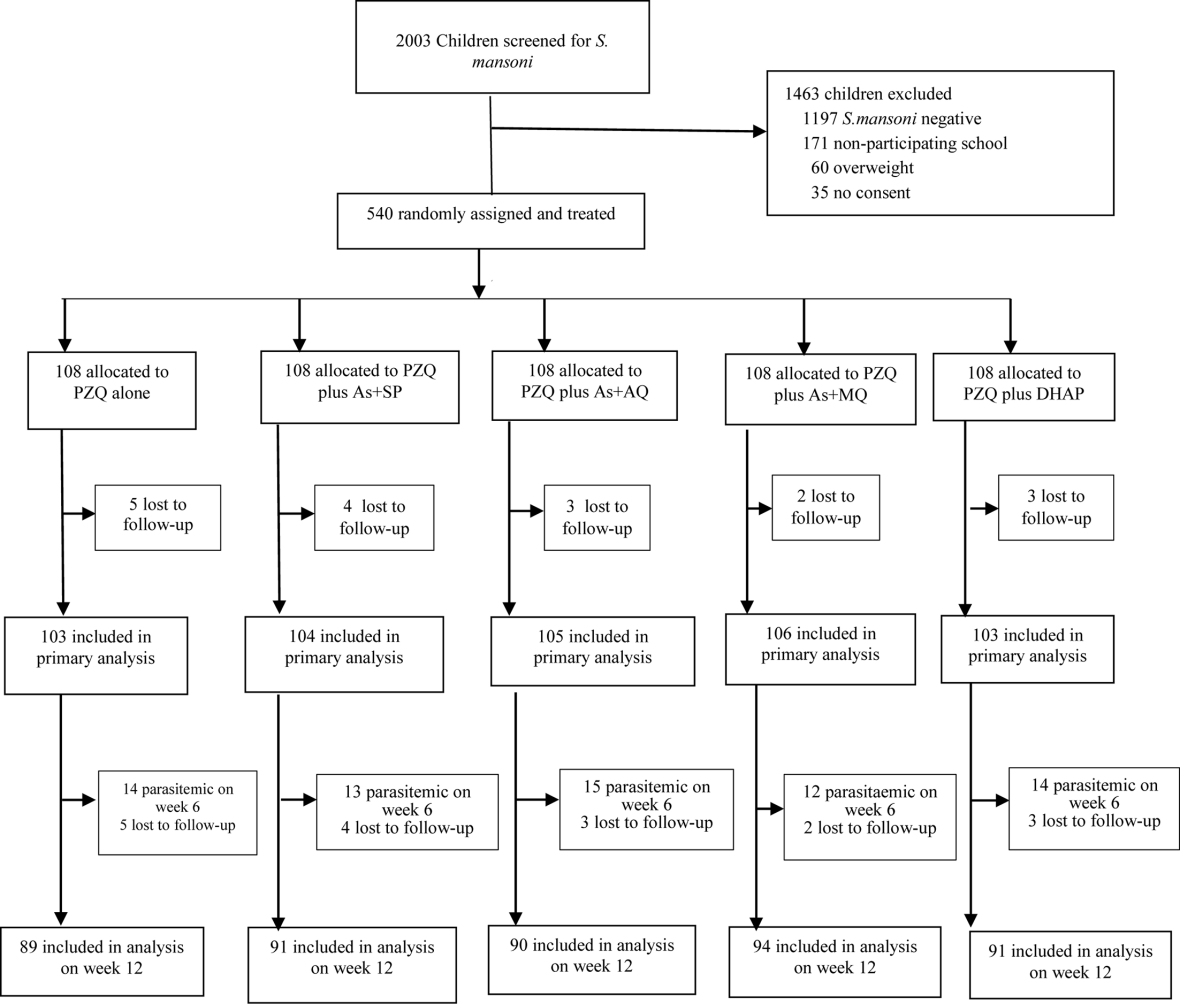
Study profile. As + AQ = artesunate plus amodiaquine; As + MQ = artesunate plus mefloquine; As + SP = artesunate plus sulfalene/pyrimethamine; DHAP = dihydroartemisinin-piperaquine; PZQ = praziquantel.

A total of 2,003 children were screened for *S. mansoni* infection, of whom 806 (40.2%) tested positive for *S. mansoni*, and none tested positive for malaria. Five hundred forty children were enrolled and randomized into five study groups (Fig. 1). After randomization, 17 (3.1%) children were lost to follow-up, and another 85 (15.7%) were lost to follow-up between Weeks 6 and 12.

The mean age of enrolled children was 10.6 (SD 1.4) years, and 47.8% (258/540) were female. Overall, the intensity of infection was light 344 (63.7%), moderate 140 (25.6%), or heavy 54 (10.4%). Treatment arms were balanced regarding age, sex, body weight, and baseline infection intensity. Table 1 is a summary of the baseline characteristics.

**TABLE 1:** Baseline characteristics of the study participants^*a*^

Variable	PZQ alone	PZQ plus As + SP	PZQ plus As + AQ	PZQ plus As + MQ	PZQ plus DHAP
Number	108	108	108	108	108
Sex					
Male, *N* (%)	54 (50.0)	61 (56.5)	60 (55.6)	54 (50)	53 (49.1)
Female, *N* (%)	54 (50.0)	47 (43.5)	48 (44.4)	54 (50)	55 (50.9)
Age (years)					
Mean (SD)	10.6 (1.3)	10.6 (1.4)	10.6 (1.4)	10.6 (1.4)	10.5 (1.4)
Age group					
<12 years, *N* (%)	82 (75.9)	76 (70.4)	76 (70.4)	77 (71.3)	83 (76.9)
≥12 years, *N* (%)	26 (24.1)	32 (29.6)	32 (29.6)	31 (28.7)	25 (23.1)
Primary schools					
Thiba, *N* (%)	14 (13.0)	16 (14.8)	13 (12.0)	13 (12.0)	15 (13.9)
Mbui-Njeru, *N* (%)	16 (14.8)	17 (15.7)	13 (12.0)	16 (14.8)	17 (15.7)
Mukou, *N* (%)	19 (17.6)	18 (16.7)	20 (18.5)	21 (19.4)	20 (18.5)
Mianya, *N* (%)	20 (18.5)	18 (16.7)	19 (17.8)	18 (16.7)	17 (15.7)
Nyangati, *N* (%)	10 (9.3)	9 (18.3)	11 (10.2)	10 (9.3)	10 (9.3)
Murubara, *N* (%)	18 (16.7)	18 (16.7)	20 (18.5)	19 (17.6)	18 (16.7)
Kamuchege, *N* (%)	11 (10.2)	12 (11.1)	12 (11.1)	11 (10.2)	11 (10.2)
Class					
3, *N* (%)	32 (29.6)	32 (29.6)	30 (27.8)	29 (26.9)	30 (27.8)
4, *N* (%)	38 (35.2)	38 (35.2)	33 (30.6)	41 (38.0)	36 (33.3)
5, *N* (%)	38 (35.2)	38 (35.2)	45 (41.7)	38 (35.2)	42 (38.9)
Body weight (kg)					
Mean (SD)	29.5 (5.5)	29.2 (4.7)	30.6 (5.8)	30.6 (6.1)	29.9 (5.9)
Hemoglobin (g/dL)					
Mean (SD)	13.4 (1.1)	13.2 (1.1)	13.3 (1.1)	13.2 (1.2)	13.2 (1.1)
Infection intensity					
Light, *N* (%)	59 (54.5)	70 (64.8)	72 (66.7)	67 (62.0)	76 (70.4)
Moderate, *N* (%)	30 (27.8)	27 (25.0)	25 (23.1)	34 (31.5)	24 (22.2)
Heavy, *N* (%)	19 (17.6)	11 (10.2)	11 (10.2)	7 (6.5)	8 (7.4)
Egg count on Day 0					
Arithmetic mean egg count (epg, 95% CI)	255.2 (166.8–343.4)	294.9 (122.9–466.8)	204.2 (104.8–303.6)	162.7 (99.2–226.1)	136.9 (81.7–192.1)

^*a*^ Key: N = number; CI = confidence interval; SD = standard deviation; PZQ = praziquantel; As + SP = a rtesunate plus sulfalene/pyrimethamine; As + AQ = artesunate plus amodiaquine; As + MQ = artesunate plus mefloquine; DHAP = dihydroartemisinin-piperaquine.

Five hundred twenty-three (96.9%) of the 540 children included were analyzed for the primary outcome, and 434 (80.4%) were cured. A cure was attained in 82.5%, 81.7%, 76.2%, 88.7%, and 85.7% of children who received PZQ alone, PZQ plus As + SP, PZQ plus As + AQ, PZQ plus As + MQ, and PZQ plus DHAP, respectively. Non-inferiority was declared for PZQ plus As + MQ (difference 6.2 [95% confidence interval, CI: −3.3 to 15.6]) and PZQ plus DHAP (3.2 [−6.7 to 13.1]) but not for PZQ plus As + SP or PZQ plus As + AQ. Across all treatment groups, there was an inverse relationship between cure rates and the intensity of pre-treatment infection (Table 2).

**TABLE 2:** Summary of primary efficacy endpoints^*a*^

Variable	PZQ alone	PZQ plus AS + SP	PZQ plus As + AQ	PZQ plus As + MQ	PZQ plus DHAP
Cure rate on Week 6 [PP],	85/103 (82.5%)	85/104 (81.7%)	80/105 (76.2%)	94/106 (88.7%)	90/105 (85.7%)
*n*/*N* (%) [95% CI]	[75.2–89.5%]	[74.3–89.1%]	[68.1–84.3%]	[82.7–94.7%]	[79.0–92.4%]
Risk ratio (95% CI)	Ref	0.99 (0.87–1.12)	0.92 (0.80–1.06)	1.07 (0.96–1.20)	1.04 (0.92–1.17)
Risk difference (95% CI)	Ref	−0.80 (−11.2 to 9.6)	−6.33 (−17.3 to 4.6)	6.15 (−3.3 to 15.6)	3.19 (−6.74 to 13.1)
Cure rate on Week 6 [ITT]	85/108 (78.7%)	85/108 (78.7%)	80/108 (74.1%)	94/108 (87.0%)	90/108 (83.3%)
n/N (%) [95% CI]	[69.9–85.5%]	[69.9–85.5%]	[64.9–81.5%]	[79.2–92.2%]	[75.0–89.3%]
Risk ratio (95% CI)	Ref	1.0 (0.87–1.14)	0.94 (0.81–1.09)	1.11 (0.98–1.25)	1.06 (0.93–1.21)
Risk difference (95% CI)	Ref	0 (−10.9 to 10.9)	−4.6 (−15.9 to 6.68)	8.3 (−1.65 to 18.3)	4.6 (−5.8 to 15.1)
Cure rate by baseline infection intensity					
Light	50/56 (89.3)	61/67 (91.0)	60/69 (87.0)	59/66 (89.4)	66/73 (90.4)
Moderate	24/29 (82.8)	18/26 (69.2)	15/25 (60.0)	28/33 (84.8)	20/24 (83.3)
Heavy	11/18 (61.1)	6/11 (54.5)	5/11 (45.5)	7/7 (100)	4/8 (50)

^*a*^ Key: PZQ = praziquantel; As + SP = artesunate plus sulfalene/pyrimethamine; As + AQ = artesunate plus amodiaquine; As + MQ = artesunate plus mefloquine; DHAP = dihydroartemisinin-piperaquine; n = number; N = total number; CI = confidence interval; ITT = intention-to-treat; PP = per-protocol; Ref = reference.

From the mITT, the corresponding cure rates were 78.7%, 78.7%, 74.1%, 87.0%, and 83.3%, respectively. Non-inferiority was also declared for PZQ plus As + MQ (8.3 [95% CI: −1.7 to 18.3]) and PZQ plus DHAP (4.6 [95% CI: −5.8 to 15.1]), but not for PZQ plus As + SP or PZQ plus As + AQ (Table 2).

**Fig 2:**
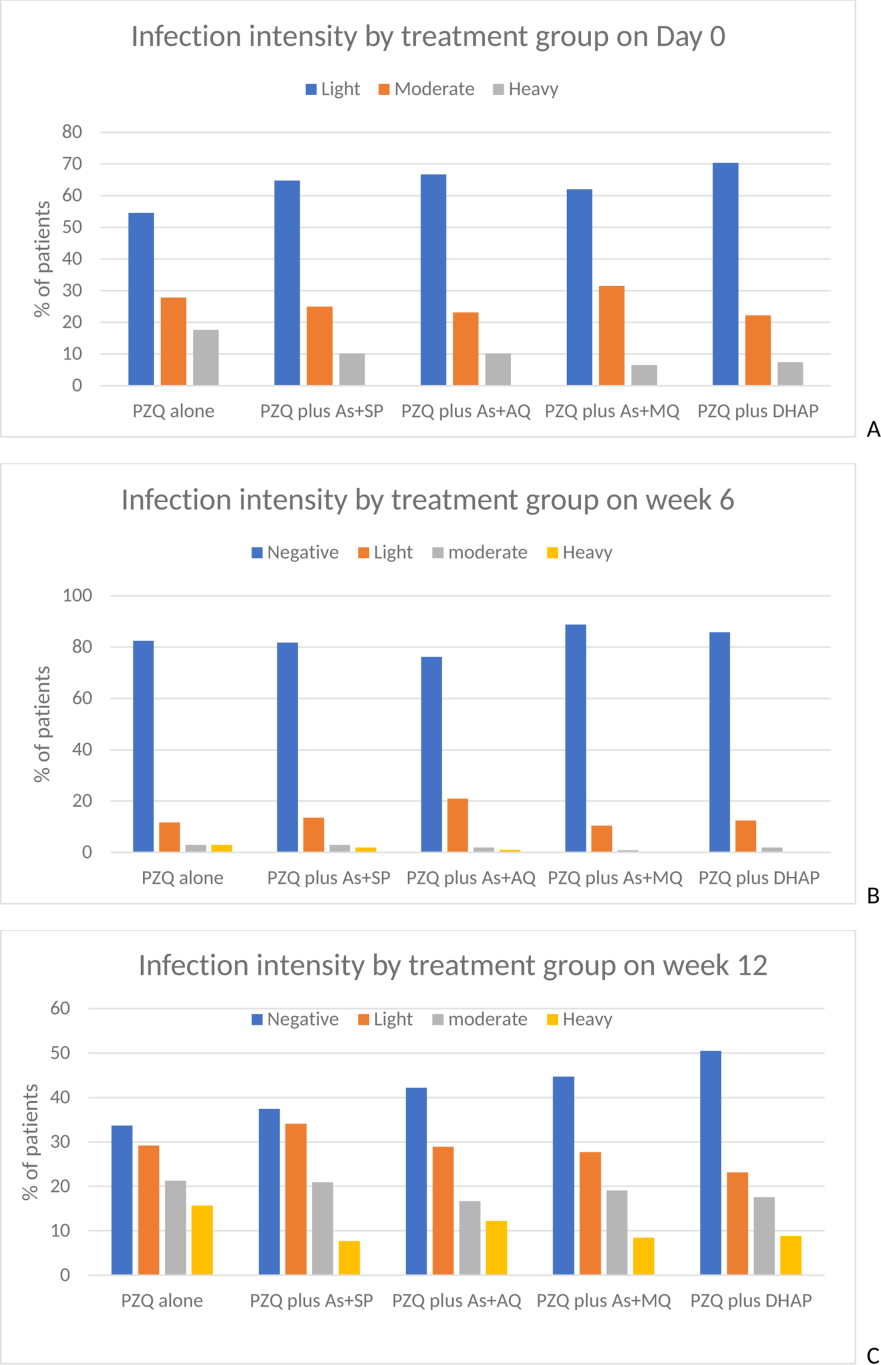
Changes in infection intensity at baseline, 6, and 12 weeks. PZQ = praziquantel; As + SP = artesunate plus sulfalene/pyrimethamine; As + AQ = artesunate plus amodiaquine; As + MQ = artesunate plus mefloquine; DHAP = dihydroartemisinin-piperaquine; A = at baseline; B = at 6-week follow-up; C = at 12 weeks after treatment.

The ERR at 6 weeks was 84.2% with PZQ alone but above 90% in the four combination treatment arms (Table 3). The mean egg excretion was significantly increased on PZQ plus As + MQ and PZQ plus DHAP arms but not on the PZQ plus As + SP or PZQ plus As + AQ arms (Table 3). Across all the combination therapy groups, infection intensity decreased significantly relative to the PZQ group (Fig. 2, panel B).

**TABLE 3:** Summary of secondary endpoints^*a*^

	PZQ alone	PZQ plus AS + SP	PZQ plus As + AQ	PZQ plus As + MQ	PZQ plus DHAP
Cure rate on Week 12,	59/89 (66.3)	57/91 (62.6)	52/90 (57.8)	52/94 (55.3)	45/91 (49.5)
*n*/*N* (%) [95% CI]	[55.8–75.4%]	[52.2–72.0%]	[45.1–65.1%]	[39.2–59.7%]	[47.3–67.6%]
Risk ratio (95% CI)	Ref	0.94 (0.76–1.17)	0.87 (0.69–1.09)	0.83 (0.66–1.05)	0.75 (0.58–0.96)
Arithmetic mean EPG					
Baseline EPG (95% CI)	255.2 (166.8–343.4)	294.9 (122.9–466.8)	204.2 (104.8–303.6)	162.7 (99.2–226.1)	136.9 (81.7–192.1)
Mean difference in egg count at 6 weeks, Δ, (95% CI), *P* value	Ref	25.8 (−3.7 to 55.3), 0.09	24.5 (−4.9 to 53.9), 0.10	35.5 (6.2–64.9), 0.02	35.7 (6.3–65.1), 0.02
Mean EPG at 6 weeks (95% CI)	40.3 (1.19–81.7)	14.5 (1.83–27.2)	15.8 (2.14–33.7)	4.73 (0.43–9.91)	4.57 (0.64–8.49)
Egg reduction rate (%)^1^	84.2 (77.2–91.2)	95.1 (90.9–99.2)	92.2 (87.1–97.3)	97.1 (93.9–00)	96.7 (93.2–100)
Mean difference in egg count at 12 weeks, Δ, (95% CI), *P* value	Ref	104.1 (−5.1 to 213.2), 0.06	62.1 (−47.4 to 171.5), 0.27	82.6 (−25.8 to 190.9), 0.14	111.0 (1.87–220.2), 0.046
Mean EPG at 12 weeks (95% CI)	234.3 (130.9–337.8)	130.29 (64.7–195.9)	172.27 (98.4–246.1)	151.78 (70.6–232.9)	123.29 (66.6–179.9)
Egg reduction rate (95% CI)^2^	8.2 (2.50–13.9)	55.8 (45.6–66.0)	15.6 (8.10–23.1)	6.7 (1.65–11.8)	9.9 (3.16–16.0)
Incidence of adverse events					
No. of children with adverse events, *N* (%)	15 (13.9)	35 (32.4)	29 (26.9)	31 (28.7)	28 (25.9)
Risk ratio (95% CI)	Ref	2.33 (1.39–3.91)	1.93 (1.12–3.34)	2.07 (1.21–3.53)	1.87 (1.07–3.24)

^*a*^ PZQ = praziquantel; As + SP = artesunate plus sulfalene/pyrimethamine; As + AQ = artesunate plus amodiaquine; As+MQ = artesunate plus mefloquine; DHAP = dihydroartemisinin- piperaquine; n = number; N = total number; CI = confidence interval; Δ = mean difference; EPG = eggs per gram; Ref = reference;1 = egg reduction rate at 6 weeks; 2 = egg reduction rate at 12 weeks.

At Week 12, 455 (84.3%) children were analyzed, and 265 (58.2%) were cured. The cumulative cure rates were much lower than at Week 6 but were comparable between the treatment groups, except for the PZQ plus DHAP group, which was significantly lower than the PZQ alone (RR = 0.75, 95% CI: 0.58–0.96) (see Table 3).

One hundred ninety (43.4%) children were excreting schistosome eggs at Week 12. The mean egg counts had increased to pre-treatment levels (Fig. 2, panel C). In three combination therapy groups (PZQ plus As + AQ, PZQ plus As + MQ, and PZQ plus DHAP), the ERRs decreased significantly compared to the PZQ alone group. There was a significant increase in ERR on the PZQ plus As + SP group relative to the PZQ group, 55.8% versus 8.2% (see Table 3).

We did not observe any serious adverse events. One hundred thirty-eight (25.6%) of the 540 children reported adverse events. A significantly higher proportion of those who received combination therapy reported an adverse event than those who received PZQ alone (see Table 3). Overall, 197 adverse events were reported. The majority of the adverse events were mild (81.2%) or moderate (18.8%), with the most common being abdominal pain (20.7%), headache (7.6%), and vomiting (3.1%). All adverse events did not require any treatment. Table 4 summarises the adverse events reported by the treatment group.

**TABLE 4:** Summary of adverse events reported during the study^*a*^

Adverse event	Severity	PZQ alone	PZQ plus As + SP	PZQ plus As + AQ	PZQ plus As + MQ	PZQ plus DHAP	Total
Abdominal pain	Mild	11	19	22	25	17	94
	Moderate	2	5	3	2	6	18
Headache	Mild	2	10	9	5	5	31
	Moderate	0	2	1	3	4	10
Vomiting	Mild	0	3	4	2	4	13
	Moderate	0	1	3	0	0	4
Dizziness	Mild	1	1	2	1	1	6
	Moderate	0	2	0	0	1	3
Cough	Mild	1	1	0	0	1	3
	Moderate	0	1	0	0	0	1
Nausea	Mild	0	3	2	2	1	8
	Moderate	0	0	0	0	0	0
Runny nose	Mild	0	1	1	0	0	2
	Moderate	0	0	0	0	0	0
Chest pain	Mild	0	0	0	1	0	1
	Moderate	0	0	0	0	0	0
Anorexia	Mild	0	0	0	1	0	1
	Moderate	0	0	0	0	0	0
Diarrhoea	Mild	0	0	0	1	0	1
	Moderate	0	0	0	0	0	0
Body rash	Mild	0	0	0	0	0	0
	Moderate	0	0	1	0	0	1
		17	49	48	42	40	197

^*a*^ PZQ = praziquantel; As + SP = artesunate plus sulfalene/pyrimethamine; As + AQ = artesunate plus amodiaquine; As + MQ = artesunate plus mefloquine; DHAP = dihydroartemisinin-piperaquine.

## DISCUSSION

This study assessed the efficacy and safety of four combination therapies relative to PZQ in treating African school-aged children with *S. mansoni* infection. Two combination therapies were non-inferior to PZQ at 6 weeks post-treatment. These were PZQ plus As + MQ and PZQ plus DHAP. Compared to PZQ alone, combination therapy was associated with a significant reduction in egg excretion and ERRs above 90%, indicating satisfactory treatment efficacy. Most of our participants had light-intensity infections at enrollment and were more likely to be cured regardless of the treatment arm. The study drugs were well-tolerated, and no serious adverse events were reported. However, a greater number of children on combination therapy reported mild adverse events than children on PZQ monotherapy. The higher cure and egg reduction rates at Week 6 were not sustained through Week 12, suggesting the need for repeat dosing at prespecified intervals.

Single oral PZQ therapy is moderately effective and safe in treating intestinal schistosomiasis in our setting of moderate *S.mansoni* transmission (40% prevalence). The cure rate was 82.5%, and the ERR was 84.2% 6 weeks after treatment, consistent with a meta-analysis that found cure rates of 76.7% and ERR of 85.5% with PZQ treatment (28). Almost 90% of the children cured after treatment with PZQ alone had light-intensity *S. mansoni* infections, consistent with the observation that the cure rate is inversely associated with pre-treatment infection intensities (29). In agreement with the fear of declining PZQ efficacy, we found ERR below 90% after PZQ treatment, indicating a doubtful treatment efficacy according to the WHO criteria (30).

Our study assessed the efficacy and safety of four different combination therapies using a non-inferiority, multi-arm parallel-group design. We found that combination therapy using a standard PZQ dose and antimalarial treatment regimens over 3 days had potential benefits. Notably, combination therapy significantly reduced the intensity of infection but not its prevalence. Non-inferiority was established for PZQ plus As + MQ and PZQ plus DHAP, indicating their potential as alternatives to PZQ monotherapy. Treatment with these two combination therapies led to a significant reduction in egg excretion and an ERR above 90%, meeting the WHO criteria for satisfactory treatment efficacy (30). These findings suggest that PZQ plus As + MQ or PZQ plus DHAP could provide ancillary benefits on schistosomiasis-related morbidity reduction compared to PZQ alone. Treatment-associated reduction in infection intensity is considered a measure of treatment impact as it strongly correlates with improvements in schistosomiasis-related morbidity (31).

Our study presents new findings and, in some instances, contrasts previous research. We found no difference in cure rate between PZQ plus DHAP and PZQ alone (85.7% vs. 82.5%) at 6-week follow-up. This finding contrasts a study in Tanzania where PZQ + DHAP had superior efficacy to PZQ alone (81.9% vs. 63.9%) in children with *S. mansoni* infections at 8-week post-treatment (18). However, the Tanzanian study is labeled as a non-inferiority trial, but the report shows no indication that it was designed and analyzed as a non-inferiority study. DHAP is an excellent antimalarial with potential benefits for malaria treatment and prevention (as seasonal malaria chemoprophylaxis and intermittent preventive treatment of malaria in school-aged children and pregnant women) in the same population groups at risk of schistosomiasis. A combination therapy using PZQ plus DHAP will likely be a feasible, cost-effective intervention targeting the treatment, prevention, and control of both schistosomiasis and malaria.

We found comparable cure rates with PZQ plus As + MQ relative to PZQ (88.7% vs. 82.5%) at 6-week post-treatment. The result contrasts a study in Cote d’Ivoire where relatively low cure rates (33% vs. 19%) were observed at 78–79 days (11 weeks) after treatment of children with *S. haematobium* using PZQ plus As + MQ compared to PZQ (19). These differences in response rates can be explained by the differential sensitivity of the schistosome species to treatment. As + MQ is a safe and effective ACT that has the potential for the treatment, chemoprevention, and control of both schistosomiasis and malaria in school-aged children and pregnant women.

In this study, the finding of an inconclusive cure rate with PZQ plus As + SP and PZQ plus As + AQ relative to PZQ alone was surprising. The ERR of 95% and 92.2% for PZQ plus As + SP and PZQ plus As + AQ, respectively, relative to 84.2% for PZQ alone, indicates satisfactory efficacy, but the assessment by cure rate was inconclusive. However, compared to PZQ alone, these combination therapies were not associated with a significant reduction in egg excretion post-treatment. These findings suggest that these two combinations should be excluded from further assessment of combination therapy for intestinal schistosomiasis.

The study drugs were generally well tolerated, with most adverse events being mild and transient. Overall, 26% of the participants reported an adverse event, with most participants reporting abdominal pain. PZQ was the most tolerable treatment, consistent with prior PZQ studies (32, 33). However, a higher proportion of children on combination treatment reported adverse events. Previous studies that assessed ACT compared to PZQ for treating participants with schistosomiasis found fewer adverse events in the ACT arm (22–27). The relative safety of the study drugs administered separately suggests that sequential, rather than simultaneous, administration may improve the safety outcomes. Our finding of a higher frequency of adverse events with combination therapy is consistent with a study in Cote d’Ivoire where 91% versus 50% of children treated with PZQ plus As + MQ reported adverse events relative to those who received PZQ alone (19). By contrast, PZQ plus DHAP was relatively safe in Tanzania, where comparable proportions of children (33.6% vs. 28.4%) reported adverse events compared to those who received PZQ alone (18). Drug-drug interactions from the simultaneous drug administration and the higher pill burden could explain the high risk of adverse events in the combined therapy groups. The use of self-reports, limited sample size, and short duration of the study limited our ability to compare drug safety confidently. The number of adverse events observed with combination therapy calls for great caution and close safety monitoring in future studies of artesunate-based combination therapies for schistosomiasis.

Several limitations to the study were identified. Most notably, our study may have been underpowered due to an inadequate sample size. Most children had light-intensity infections at baseline; thus, the findings do not represent high transmission settings. A double-blind design could have been beneficial, but including matching placebo tablets for five different drugs was not feasible. For logistical reasons, single stool samples were used to diagnose *S. mansoni*, which may have led to an overestimation of the cure rate or misclassification in some children due to the Kato-Katz method’s lack of sensitivity in low-intensity infections. Moreover, the study did not directly measure the treatment effect on juvenile worms, and it is uncertain whether 6-week post-treatment is the best time to measure efficacy. It is challenging to compare findings since studies have evaluated cure rates at various times due to the lack of approved testing protocols for schistosomiasis. Participants on combined therapy received a 3-day course of treatment, while those on the reference treatment arm received a single oral dose. These differences could impact on compliance and the reporting of adverse events. Finally, due to the short follow-up period, the study failed to assess the long-term safety and efficacy outcomes.

Our study raises important questions about the safety, effectiveness, and interactions of drugs used in combination therapy for intestinal schistosomiasis and offers potential solutions. Overall, combination therapy (PZQ plus ACT) is a promising strategy for improving the treatment, prevention, and control of schistosomiasis in endemic areas. There are a few unresolved issues regarding the dosing intervals, sequential versus simultaneous administration, and the relative advantage between ERR and cure rate as the best measure of treatment efficacy in schistosomiasis treatment studies. Our study suggests that ERR may be a better measure of treatment efficacy than cure rate (34, 35). However, further research is needed to validate these findings across schistosome species in different epidemiological settings and populations. Future studies should monitor changes in the prevalence of malaria and the frequency of molecular markers of artemisinin drug resistance following combination therapy for schistosomiasis. Parasitic disease control programs should assess the trends in the prevalence and intensity of schistosomiasis in malaria-schistosomiasis co-endemic areas since the introduction of ACTs for malaria control. We recommend that combination therapy (PZQ plus ACT) could be a promising strategy for seasonal malaria chemoprophylaxis for preschool and school-aged children in areas where malaria and schistosomiasis coexist, in the prevention of malaria (and schistosomiasis) in pregnancy, in patients with both malaria and schistosomiasis, as a backup treatment for PZQ failure, and as treatment for returning travelers from schistosomiasis-endemic areas. If the safety and effectiveness of combination therapy are confirmed, it meets the criteria set for desirable qualities of alternative new anti-schistosomal treatments: high efficacy against all stages of the worm, effective against all the main human schistosome species, can be used by all individuals at-risk (including pregnant women), once or twice daily oral dosing, minor, manageable side effects, use in pregnant women and infants, and no clinically significant drug-drug interactions (36). In conclusion, we found that PZQ plus As + MQ and PZQ plus DHAP were non-inferior to PZQ and were effective in reducing infection intensity. However, these results should be confirmed across schistosome species in different population groups (preschool and pregnant women) and epidemiological settings.

## MATERIALS AND METHODS

### Study design and participants

This was a randomized, open-label, five-arm, head-to-head, non-inferiority trial. We enrolled children (aged 9–15 years) from seven primary schools with a high prevalence of schistosomiasis within the Mwea irrigation scheme in Kirinyaga County, central Kenya. The children were enrolled and treated between September 2018 and January 2019. The study area is endemic for *S. mansoni,* with up to 75% prevalence in school children, and has extremely low malaria transmission (<1/1,000 individuals) (37). The major socio-economic activities in the study area are rice growing under irrigation and small-scale subsistence farming. The protocol for this study is published elsewhere (38).

Before starting the study, a meeting was held with parents, teachers, and community leaders to inform them about the study’s objectives, procedures, and potential benefits and risks. The County Commissioner, the Directorate of Health and Education, and the respective school administration permitted the study. To be included in the study, the children had to have parasitologically confirmed *S. mansoni* infection, be able to take oral treatment and be willing to participate in follow-up evaluations. Children were excluded if they weighed more than 50 kg, were pregnant, had hemoglobin level ≤8.0 g/dL, had co-infection with *Plasmodium falciparum*, had severe malnutrition, had a history of convulsions or allergy to artemisinin-based antimalarials, sulfonamides, mefloquine or praziquantel, or had received treatment with an antimalarial or anti-schistosomal drug within a month before joining the study.

### Randomization and masking

Children were randomly assigned (1:1:1:1:1) to receive praziquantel with artesunate plus sulfalene-pyrimethamine, praziquantel with artesunate plus amodiaquine, praziquantel with artesunate plus mefloquine, praziquantel with dihydroartemisinin-piperaquine or praziquantel alone. The randomization sequence (in blocks of 15) was computer-generated by an independent statistician. The individual randomization numbers and accompanying treatment assignment were stored in sequentially numbered opaque sealed envelopes. The envelopes were assigned by the study nurse in sequential order to participants at enrolment. The laboratory technicians were masked to the treatment assignment. However, participants, the study clinicians, and nurses were not masked to the treatment groups. Participants and the study nurses could have noticed the different treatments because of the differences in packaging, shapes, size, and number of tablets.

### Procedures

The participants were recruited from the participating schools. Children provided fresh stool samples, which were screened for eggs of *S. mansoni* and soil-transmitted helminth. The study clinician took a brief medical history and performed a physical examination to confirm the eligibility of every child. The children whose stools tested positive for *S. mansoni* and who met the selection criteria were enrolled in the study.

At enrollment, the study clinician took a standard baseline medical history and conducted a clinical examination, including assessing the liver and spleen size, weight, and height measurements. A capillary blood sample was taken from a fingerprick to estimate hemoglobin and to detect the presence of malaria parasites. The clinician sequentially assigned study identification numbers at enrolment. Study data were recorded on case record forms. Participants were identified using their initials and unique study identification numbers during the study.

All participants received a single dose of praziquantel (PZQ) (Biltricide, Bayer Healthcare, Leverkusen, Germany) at 40 mg/kg to the nearest half tablet (600 mg tablet). In addition, participants assigned to the praziquantel with artesunate plus sulfalene-pyrimethamine (As + SP) arm received a 3-day course of artesunate-sulfalene-pyrimethamine (Coarinate Junior FDC, Dafra Pharma, Turnhout, Belgium) tablets as 4 mg/kg/day of the artesunate component once daily for 3 days. As + SP is a fixed-dose combination of 100 mg artesunate, 250 mg sulfalene, and 12.5 mg pyrimethamine (pack of three tablets). Participants assigned to the praziquantel with artesunate plus amodiaquine (As + AQ) arm received a 3-day course of artesunate-amodiaquine (ASAQ, Winthrop, Sanofi-Aventis, France) tablets as 4 mg/kg/day of the artesunate component once daily for 3 days. As + AQ is a fixed-dose combination of 100 mg artesunate and 270 mg amodiaquine. Participants assigned to the praziquantel with artesunate plus mefloquine (As + MQ) arm received a 3-day course of artesunate plus mefloquine (ARTEQUIN, Acino/Mepha, Switzerland) tablets as 4 mg/kg/day of the artesunate and 8 mg/kg/day of mefloquine once daily for 3 days. As + MQ is a fixed-dose combination of 200 mg artesunate and 250 mg mefloquine. Participants assigned to the praziquantel with dihydroartemisinin-piperaquine (DHAP) arm received a 3-day course of dihydroartemisinin-piperaquine (D-ARTEPP, Guilin Pharmaceutical Co. Ltd, China) tablets as 4 mg/kg/day of dihydroartemisinin and 20 mg/kg/day of piperaquine once daily for 3 days. DHAP is a fixed-dose combination of 40 mg of dihydroartemisinin and 320 mg of piperaquine.

Before drug administration, all children were provided food to reduce the nauseating effect of the study drugs and improve their bioavailability (39). The study nurse supervised the oral administration of all the study drugs. Children were observed for 1-h post-treatment for acute adverse events. If vomiting occurred within 1 h of drug ingestion, a repeat dose was given. All children with soil-transmitted infections received a single dose of 400 mg of albendazole. Concomitant drugs were prescribed as needed and recorded on the case record forms. The use of drugs with antimalarial activity was not permitted in the study.

Participants were followed up for a total of 12 weeks. Children were visited at school or home during the first 3 days after enrollment to administer the Day 2 and 3 treatment and complete the adverse events questionnaire. The primary outcomes were assessed 6-week post-treatment, a time point corresponding with the duration of schistosome larval maturation. At the Week 6 and 12 follow-up visits, the participants provided an early morning stool sample, and the study clinician took a standardized medical history, performed a clinical examination, and obtained a fingerprick blood sample (for hemoglobin estimation). Participants who were still parasitaemic at Week 6 were treated with a single dose of 40 mg/kg of praziquantel and withdrawn from the study. Those who were unable to retain study medication (due to repeated vomiting), withdrew consent, were lost to follow-up, developed a serious adverse event, or had a major protocol violation (such as missing more than one treatment dose or self-medication with an antimalarial or anti-schistosomal drug) were also withdrawn from the study. At the end of the study, all participants were treated with a single dose of 40 mg/kg of PZQ.

All the laboratory tests were performed by the laboratory at the Kimbimbi sub-County hospital, using standard techniques. Stool samples from children at enrolment, Weeks 6 and 12 were assessed. All stool samples’ appearance (color, consistency, and presence of worms, blood, mucus, or pus) was recorded. Duplicate slides from one stool sample were prepared and examined independently under the microscope by two skilled laboratory technicians. The *S. mansoni* egg and soil-transmitted helminth egg were quantified using the Kato-Katz fecal smear technique (40). A template containing approximately 41.7 mg feces was filled, the number of *S. mansoni* eggs per slide was counted, and the mean of the two slides was multiplied by 24 and expressed as eggs per gram of feces (epg). The infection intensity was classified as light (1–99 epg), medium (100–399 epg), or heavy (>400 epg) (41). A third technician read 10% of the slides and all slides where the initial readings varied between the two technicians by more than 20%. A capillary blood sample was taken from a fingerprick to estimate hemoglobin (HemoCue) and to detect the presence of malaria parasites (by microscopy). Thin and thick blood films were Giemsa stained, air-dried, and examined for malaria parasites; to calculate malaria density, the parasites, and white blood cells were counted in the same field until 300 cells were counted.

The primary treatment outcomes were parasitological cure rate, defined as the proportion of children not shedding eggs and the incidence of adverse events between those who received combination therapy and those who received PZQ alone at Week 6 post-treatment. Secondary outcomes were an egg reduction rate (ERR) at 6 and 12 weeks and a cumulative cure rate at Week 12. The arithmetic mean egg count (AM) was calculated from the baseline and post-treatment data. The ERR was defined as the percentage reduction in the mean number of *S. mansoni* eggs between baseline and post-treatment and was calculated as 1 − [AM egg count post-treatment/AM egg count at enrollment] × 100. The level of treatment efficacy was categorized on the basis of the WHO thresholds for ERR, whereby a point estimate of ERR ≥90% is considered satisfactory, <80% is considered reduced, and ERR of between 80% and 90% is considered doubtful (30). Safety was assessed as the incidence of any adverse events elicited using an adverse events form administered after an hour of ingesting study treatment and 24 hours of such treatment (before the next ACT dose). An adverse event was defined as a sign, symptom, intercurrent illness, or abnormal laboratory finding that was not present at enrollment but occurred during follow-up. An adverse event was classified as serious if they were lethal or life-threatening, disabling, or resulted in prolonged hospital admission. Adverse events were assessed by the study clinician and graded as mild, moderate, or severe, according to their intensity as perceived by the participant and their impact on daily activities.

### Statistical analysis

We hypothesized that combination therapy was not inferior to PZQ in treating schistosomiasis and set a non-inferiority margin at −10 percentage points for the risk difference in cure rates. We assumed a cure rate of 72% with PZQ from longitudinal studies in western Kenya (42). With this non-inferiority margin, we calculated that after accounting for a dropout rate of 5%, we would enroll 108 participants in each treatment group for the study to have an 80% power at a one-sided alpha value of 0.025 without multiplicity adjustment. Therefore, the five study arms needed a total sample size of 540.

Data were double-entered into computers using MS Access (Microsoft 2010) and analyzed using IBM SPSS for Windows version 20.0 and STATA version 12.0. Baseline characteristics and outcomes data were analyzed for all children who received at least one dose of the study drug, whether they completed the study or not. For this exploratory study, adjustment for multiple comparisons was not deemed appropriate (43).

Cure rates (CRs) were compared using Pearson’s *Χ*^2^ test. The results were summarized as risk ratios (RRs) or risk differences with 95% CIs. The risk ratio was calculated as the proportion of participants cured on the combination therapy arm divided by the proportion cured on the PZQ alone arm. Risk differences with 95% CI were computed between children who received combination therapy and those who received PZQ alone. Non-inferiority was determined if the lower bound of the 95% CI for the risk difference exceeded −10. The primary outcome was analyzed using the per-protocol and modified intention-to-treat (mITT) approaches, with per-protocol analysis considered the primary approach. The per-protocol analysis was performed on the available case population, defined as all randomized participants who completed the study procedures to week six. The mITT population included all eligible participants who received at least one treatment dose. The primary outcome data were imputed for missing data. Changes in continuous variables were assessed using a one-way analysis of variance (ANOVA), and *post hoc* pairwise comparisons between group means were performed using the least significant difference. The frequency and pattern of adverse events were examined in an intent-to-treat population. Two-sided *P* values less than 0.05 were considered statistically significant without adjustments for multiple comparisons. This trial is registered with the Pan-African Clinical Trials Registry, PACTR202001919442161.

## Data Availability

The data sets used and analyzed during the current study are available from the corresponding author upon reasonable request.
